# The Level of DING Proteins Is Increased in HIV-Infected Patients: *In Vitro* and *In Vivo* Studies

**DOI:** 10.1371/journal.pone.0033062

**Published:** 2012-03-09

**Authors:** Ahmed Djeghader, Gerard Aragonès, Nune Darbinian, Mikael Elias, Daniel Gonzalez, Anabel García-Heredia, Raúl Beltrán-Debón, Rafal Kaminski, Guillaume Gotthard, Julien Hiblot, Anna Rull, Olivier Rohr, Christian Schwartz, Carlos Alonso-Villaverde, Jorge Joven, Jordi Camps, Eric Chabriere

**Affiliations:** 1 Unité de Recherche sur les Maladies Infectieuses et Tropicales Emergentes, Centre National de la Recherche Scientifique, Faculté de Médecine Aix-Marseille University, Marseille, France; 2 Centre de Recerca Biomèdica, Hospital Universitari de Sant Joan, Institut d'Investigació Sanitària Pere Virgili, Universitat Rovira i Virgili, Reus, Catalonia, Spain; 3 Department of Neuroscience, Center for Neurovirology, Temple University School of Medicine, Philadelphia, Pennsylvania, United States of America; 4 Department of Biological Chemistry, Weizmann Institute of Science, Rehovot, Israel; 5 Institut de Parasitologie et Pathologie Tropicale, Université de Strasbourg, Strasbourg, France; 6 Servei de Medicina Interna, Hospital de Son Llàtzer, Ciutat de Palma, Mallorca, Spain; New York University, United States of America

## Abstract

DING proteins constitute an interesting family, owing to their intriguing and important activities. However, after a decade of research, little is known about these proteins. In humans, at least five different DING proteins have been identified, which were implicated in important biological processes and diseases, including HIV. Indeed, recent data from different research groups have highlighted the anti-HIV activity of some DING representatives. These proteins share the ability to inhibit the transcriptional step of HIV-1, a key step of the viral cycle that is not yet targeted by the current therapies. Since such proteins have been isolated from humans, we undertook a comprehensive study that focuses on the relationship between these proteins and HIV-infection in an infectious context. Hence, we developed a home-made ELISA for the quantification of the concentration of DING proteins in human serum. Using this method, we were able to determine the concentration of DING proteins in healthy and HIV-infected patients. Interestingly, we observed a significant increase of the concentration of DING proteins in non treated and treated HIV-infected patients compared to controls. In addition, cell cultures infected with HIV also show an increased expression of DING proteins, ruling out the possible role of antiretroviral treatment in the increase of the expression of DING proteins. In conclusion, results from this study show that the organism reacts to HIV-infection by an overexpression of DING proteins.

## Introduction

DING proteins constitute an intriguing family of proteins that were named for their highly conserved N-terminal sequence DINGGG- [1], [2]. The first DING protein was discovered in humans [3], and other members have been further identified in the three kingdoms of life [4]. To date, about 40 different DING proteins have been reported, mainly in eukaryotes [5]. Despite the wide presence of these proteins, DING genes have never been identified in eukaryotes, even in the human genome [5]. Attempts to sequence eukaryotic DING genes have encountered many difficulties since no ORF or locus coding these proteins has been identified. Nevertheless, some partial nucleic sequences have been amplified from humans and plants [4] [Genbank HM171537 and Genbank HM572267 to HM572271]. Because of the lack of available genetic information, eukaryotic DING proteins sequences are very sparse and comprise, for the most of them, only some N-terminal and internal peptides [5]. This lack of sequence information has considerably hampered studies on DING proteins, but opens a large field of investigation onto this family of proteins.

In humans, at least five different DING proteins have been reported, generally identified in relation with a broad range of diseases or biological processes [4]. The sequence divergence observed between available pieces of sequences suggests that they are not products of polymorphisms, editing or splicing mechanisms. The Synovial Stimulatory Protein (SSP) was the first discovered DING protein and was hypothesized to be related to the etiology of rheumatoid arthritis [3]. The Crystal Adhesion Inhibitor (CAI) was isolated by virtue of its capacity to inhibit the formation of kidney stones [6]. Khan *et al.* reported the discovery of the Steroidogenisis-Inducing Protein (SIP) and its potent activity toward ovarian surface epithelium [7]. Recently, studies to define the resistant factor from HIV resistant cells led to identification of a new DING protein, X-DING CD4, that exhibits a human immunodeficiency virus-1 (HIV-1) inhibition activity [8]. This protein was only detectable in HIV-resistant cells, and its anti-HIV-mediated activity was blocked by specific antibodies [8]. Finally, the Human Phosphate Binding Protein (HPBP) was serendipitously discovered while performing structural studies on human paraoxonase-1 (PON1), a high-density lipoprotein (HDL) associated apolipoprotein with anti-atherogenic activity [9]. PON1 is an extensively studied protein that exhibits paraoxonase, lactonase and arylesterase activity, and participates in the antioxidant defense system [10], [11]. HPBP was found to form a very tight complex with PON1 [12], and it has been shown to be necessary for the stabilization of this protein [13], [14], [15]. The X-ray structure of HPBP has been solved [9], and the combination of crystallographic and mass spectrometry data has lead to the obtaining of the first complete sequence of a DING protein [9], [16]. Structural analysis of HPBP showed that DING proteins belong to a superfamily of high affinity phosphate binding proteins. Thus, HPBP may be considered as the first phosphate transporter identified so far in humans. However, the function related to this ability is still unknown. Recently, it has been shown that HPBP, as for X-DING CD4, has an anti-HIV activity. Interestingly, HPBP was also able to inhibit specific strains of HIV which are resistant to treatment with zidovudine (AZT), an antiretroviral drug [17].

The HIV inhibition activity within the DING family has also been described outside humans, for p27SJ and its full-length form p38SJ, a plant DING protein from *Hypericum perforatum* [18]. The biological role of this protein is not fully understood, but it is however clear that its phosphatase activity influences the cell cycle [19], [20]. Additionally, it has been shown that p27SJ/p38SJ interact with the viral protein Tat, the transcription factor C/EBP and the RNA Polymerase II to inhibit HIV proliferation [18], [20]. In fact, p27SJ as well as X-DING CD4 and HPBP inhibit HIV replication by targeting the transcription step in HIV cycle, a viral step that is not yet targeted by the currently used therapies.

HIV-infection is still a public health problem, affecting more than 33 million peoples in the world [21]. Current HIV therapies are based on the using of at least three drugs belonging to two different classes of antiretroviral drugs. Antiretroviral drugs can be divided in seven classes: a) nucleoside reverse transcriptase inhibitors (NRTIs); b) nucleotide reverse transcriptase inhibitors (NtRTIs); c) nonnucleoside reverse transcriptase inhibitors (NNRTIs); d) protease inhibitors (PIs); e) fusion inhibitors; f) co-receptor inhibitors, and g) integrase inhibitors [21], [22]. Drugs from each class target a different stage of HIV replication cycle, but none of them targets the transcription step. The discovery that some DING proteins target this step of HIV cycle opens new insights to understand the development of the disease, and might be useful to develop new anti-HIV treatment strategies. The presence in humans of two proteins with anti-HIV activity (HPBP and X-DING CD4) is intriguing and request more investigations. Thus, we carried out this study with the aim of exploring the relationship between HIV infection and the DING proteins concentration.

## Materials and Methods

### Study population

The study was performed on 207 HIV-infected patients (139 men, 68 women; mean age 38 years; range 22 to 66) attending the outpatients clinics of the Hospital Universitari de Sant Joan. Of these patients, 122 were co-infected by the hepatitis C virus (HCV). HIV-infected patients were undergoing two different treatment regimens. Fifty-two subjects were currently not receiving any type of antiretroviral therapy for at least 6 months (13 patients have never been treated and the 39 others stopped treatment more than 6 months), and 155 had received continuous treatment with an antiretroviral regimen containing zidovudine, stavudine or lamivudine (NRTIs). In addition to this antiretroviral treatment, HIV-infected patients were taking lopinavir/ritonavir (PIs; n = 71) or efavirenz (NNRTI; n = 84) for at least 6 months. The exclusion criteria were age under 18 years, renal function impairment defined as creatinine levels higher than 106 µmol/l, or presence of an AIDS-related opportunistic disease at the time of the study. The main clinical characteristics of these patients are summarized in Table S1. The control group consisted of 130 healthy volunteers who participated in an ongoing epidemiological study being conducted in our geographical area, and the details of which have been previously reported [23]. All the volunteers had been invited to attend a clinical examination and to provide a fasting blood sample. There was no clinical or analytical evidence of renal insufficiency, liver damage, neoplasia, or neurological disorders.

A fasting venous sample blood was obtained from all the participants and serum was stored at −80°C until measurements were performed. All the participants provided fully-informed written consent to participation in the study on the understanding that anonymity of all data is guaranteed. The study was approved by the Ethics Committee of the Hospital Universitari de Sant Joan de Reus (Institutional Review Board).

### Determination of serum concentration of DING proteins

Serum concentration of DING proteins was measured by in-house ELISA using rabbit polyclonal antibodies generated against HPBP. Details of the purification methods and antibody production have been previously reported [12], [24]. Because of the high sequence conservation between DING proteins, anti-HPBP antibodies are able to recognize several representatives of the DING family. As a standard, we used PfluDING (a DING protein from *Pseudomonas fluorescens* that shares 77% sequence identity with HPBP), with an initial concentration of 204 ng/ml, prepared in phosphate buffered saline (PBS). The secondary antibody was an anti-rabbit-IgG peroxidase conjugate (Invitrogen, Carlsbad, USA), diluted 1∶3,000 in PBS/1% BSA. The enzyme substrate was tetramethylbenzidine (Sigma, St. Louis, MO, USA) plus hydrogen peroxide. Tetramethylbenzidine (2 mg) was diluted in 200 µl of dimethylsulfoxide (DMSO) (Sigma, St. Louis, MO, USA) and added to 20 ml of 0.1 M citrate/acetate buffer, pH 6.0, with 72 µl urea hydrogen peroxide.

The ELISA procedure was the following: human serum samples were diluted 1∶1,000 in PBS. One hundred µl of sample or standard were added to the plate wells and incubated overnight at room temperature. After one wash with PBS, we added 150 µl of PBS/1% BSA to block the remaining absorption sites, and left for 1 hour at room temperature. After three washes, we added 100 µl of primary antibody (1∶3,000) and incubated for 1 hour at room temperature. After washing, we added 200 µl of secondary antibody, and incubated the plates again for 1 hour at room temperature. After washing, we added 200 µl of enzyme/substrate solution and incubated for 15 min. The reaction was stopped with 50 µl of 2 M H_2_SO_4_, and the absorbance was read at 450 nm in an automated microplate reader (BioTek Instruments Inc., Winooski, VT, USA). All the incubations were under gentle shaking. The assays were performed on 96-well low binding plates (Scientific Laboratory Supplies Ltd., Yorkshire, UK).

### Other biochemical and serological measurements

Serum PON1 concentration was determined by in-house ELISA with rabbit polyclonal antibodies generated against a synthetic peptide with the sequence CRNHQSSYQTRLNALREVQ that is specific of mature PON1 [25]. Serum PON1 lactonase activity was analyzed by measuring the hydrolysis of 5-thiobutyl butyrolactone (TBBL) as previously described [26], [27]. Serum PON1 paraoxonase activity was determined by measuring the rate of hydrolysis of paraoxon [28]. Plasma HIV-1 viral load was measured with the COBAS® TaqMan® HIV-1 assay (Roche, Basel, Switzerland) and CD4+ T-cell and CD8+ T cell counts by flow cytometry (Coulter Epics XL-MLC, Beckman Coulter, Fullerton, CA, USA). Antibodies against HCV, serum β-2-microglobulin, a marker of lymphocyte destruction and progression of HIV-infection [29], and serum cholesterol, triglycerides, HDL-cholesterol, and apolipoprotein A-I were measured in an automated analyzer (UniCel™ DxI 800, Beckman Coulter, Fullerton, CA, USA). LDL-cholesterol concentration was estimated by the Friedewald formula [30].

### FPLC lipoprotein fractionation

DING proteins distribution in lipoproteins was investigated by FPLC (Bio-Rad BioLogic DuoFlow 10 system, Bio-Rad Laboratories, Inc. Hercules, CA). Sera from 2 control subjects and 2 HIV-infected patients were pooled separately, 200 µl from each pool were injected into a Superose 6/300 GL column (GE Healthcare Europe GmbH, Glattbrugg, Switzerland), and 500 µl fractions were collected. DING proteins levels, cholesterol and triglycerides were analyzed in every fraction as previously described.

### Statistical analysis

The normality of distributions was determined with the Kolmogorov-Smirnov test. Differences between two groups were assessed with the Student's *t*-test (parametric) or the Mann-Whitney *U* test (non-parametric). Differences between multiple groups were analyzed by the Kruskal-Wallis test. Pearson or Spearman correlation coefficients were used to evaluate the degree of association between variables. The SPSS 18.0 package was employed for all statistical calculations. Receiver Operating Characteristics (ROC) analysis was employed to investigate the ability of DING proteins measurement to distinguish between HIV-infected patients and controls [31]. The curve was plotted using Xlstat software (Addinsoft, http://www.xlstat.com/en/home/).

### Infection of peripheral blood mononuclear cells (PBMC) with HIV-1

PBMC were maintained in modified RPMI media. PBMC were infected with the JR-FL strain of HIV-1 as follows. In all, 50 ng of p24-containing virus stock was added to every 1×10^6^ cells. Cells were incubated with virus stock in a small volume of serum-free medium for 2 h at 37°C. The cells were then washed twice PBS, and fresh medium was added. In parallel, control uninfected cells were prepared four days post-HIV-1-infection. Cells were harvested and analyzed by western-blot assay and ELISA as described below. Western blot analysis was performed with protein extracts using anti-HPBP, and anti-tubulin antibodies according to standard procedures.

### p24 ELISA

Approximately 1×10^6^ PBMC were infected with HIV-1 JF-RL. Six days post-infection, supernatants were collected and analyzed for the presence of p24 by ELISA assay using p24 ELISA Assay kit purchased from BioChain (BioChain Institute, Inc., Hayward, CA). The assay was measured spectrophotometrically using 450 nm filter to indicate the concentration of p24 reactive determinants present in samples.

## Results

### DING proteins concentration is increased in HIV-infected patients

**Figure 1:**
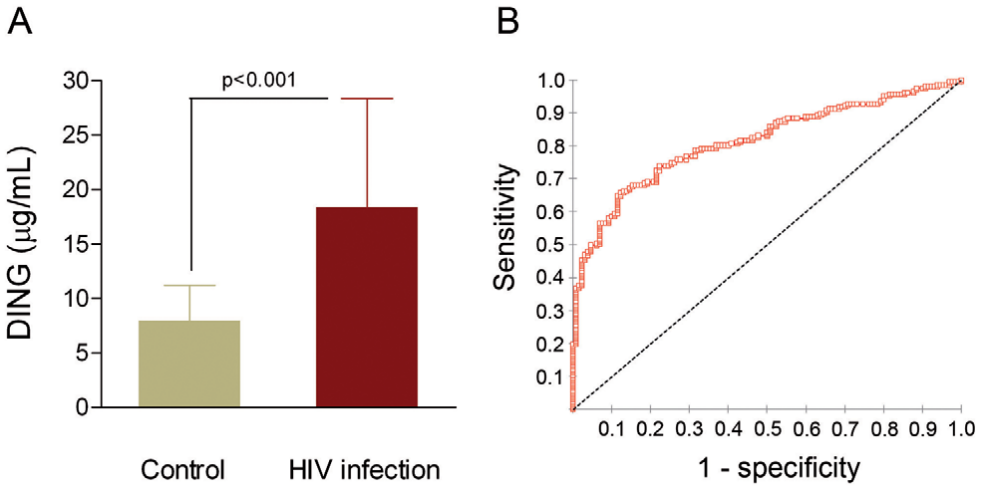
DING proteins concentrations and ROC curve obtained for the ELISA assay. (**A**) Variation of concentration between HIV-infected patients and controls; results are represented as medians and interquartile range (IQR). (**B**) ROC curve obtained for the ELISA assay; dashed line show the line of non discrimination.

Serum DING proteins concentration was significantly increased in HIV-infected patients with respect to the control group [18.3 (11.0–28.3) vs. 7.9 (4.2–11.2) mg/l, respectively; p<0.001] (Fig. 1A). The diagnostic value of our ELISA dosage was investigated using ROC curve by plotting the true positive rate (sensitivity) against the false positive rate (1- specificity). The best diagnostic test is a test in which the curve is the most closer to the upper left corner of the graph. The accuracy of the test is measured by the area under the curve (AUC) which could be different from 0.5 (non discrimination) and the closest to 1. Our ROC analysis showed an AUC of 0.813 (95%CI: 0.769 to 0.858). This value, close to 1.0 indicates that measuring DING proteins concentration may be considered as a potential predictive factor for HIV-infection (Fig. 1B).

### Relationships between serum DING proteins concentration, PON1 status and lipoprotein alterations

**Figure 2:**
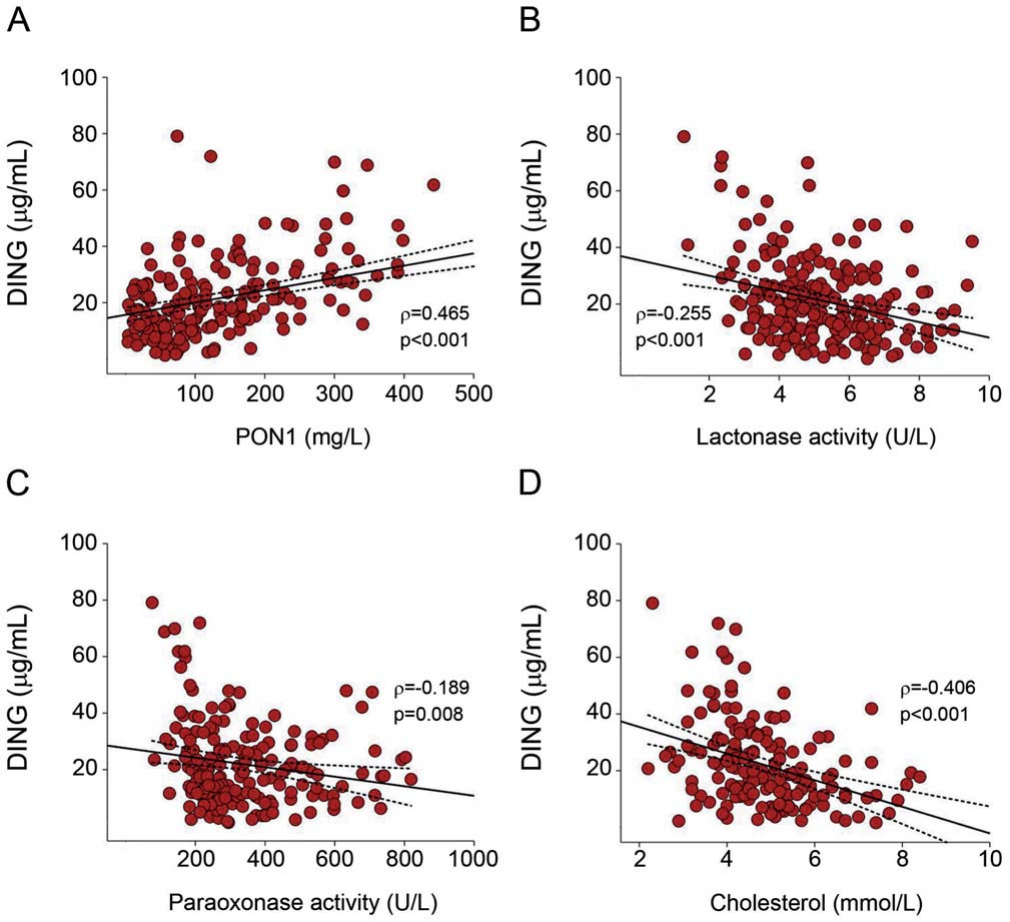
Relationship between DING proteins concentration, PON1 status and cholesterol. Correlation of DING proteins concentration with: (**A**) serum PON1 concentrations, (**B**) PON1 lactonase activity, (**C**) PON1 paraoxonase activity and (**D**) cholesterol concentration in HIV-infected patients.

Results are summarized in Fig. 2 and Table 1. HIV-infected patients had an altered lipoprotein profile with an increase in serum triglycerides concentration and a decrease in cholesterol in LDL and HDL fractions. A significant direct relationship was observed between serum DING proteins and PON1 concentrations in HIV-infected patients (*r* =  0.465; p<0.001; Fig. 2A), and an inverse relationship between DING proteins and PON1 lactonase (*r* = −0.255; p<0.001; Fig. 2B) and paraoxonase activities (*r* = −0.189; p = 0.008; Fig. 2C). DING proteins concentration was also inversely associated with serum cholesterol (*r* = −0.406; p<0.001; Fig. 2D), LDL-cholesterol (*r* = −0.310; p<0.001), HDL-cholesterol (*r* = −0.174; p = 0.016), serum triglycerides (*r* = −0.146; p = 0.050), and apolipoprotein A-I (*r* = −0.221; p = 0.002) concentrations.

**Table 1:** Selected biochemical parameters in the control group and in HIV-infected patients.

Parameter	Control group (n = 130)	HIV-infected patients (n = 207)	p*	Untreated (n = 52)	Treated (n = 155)	p†
Cholesterol (mmol/L)	5.28 (0.98)	4.89 (1.23)	<0.001	4.47 (0.93)	5.03 (1.29)	0.007
Triglycerides (mmol/L)	1.1 (0.5–2.6)	1.5 (0.6–8.5)	<0.001	1.1 (0.6–4.1)	1.8 (0.7–8.6)	<0.001
HDL-cholesterol (mmol/L)	1.48 (0.39)	1.18 (0.45)	<0.001	1.19 (0.37)	1.17 (0.46)	NS
LDL-cholesterol (mmol/L)	3.20 (0.95)	2.75 (0.96)	<0.001	2.57 (0.79)	2.82 (1.01)	NS
Apolipoprotein A-I (g/L)	1.69 (0.28)	1.38 (0.31)	<0.001	1.38 (0.31)	1.38 (0.31)	NS
PON1 concentration (mg/L)	96.5 (43.6–291)	98.9 (14.1–347)	NS	115.9 (13.7–571)	93.5 (13.7–338)	0.06
PON1 lactonase activity (U/L)	5.4 (3.2–8.8)	5.2 (2.8–8.5)	NS	4.9 (2.3–8.1)	5.1 (2.9–8.3)	NS
PON1 paraoxonase activity (U/L)	278.2 (161–580)	285.7 (153–679)	NS	275.3 (123–678)	285.9 (171–709)	NS

Results are presented as means and SD in parentheses (parametric) or as medians and 95% CI in parenthesis (nonparametric). NS: Not significant.

* Control group *versus* all HIV-infected patients.

† Treated *versus* untreated HIV-infected patients.

### FPLC lipoproteins fractionation

**Figure 3:**
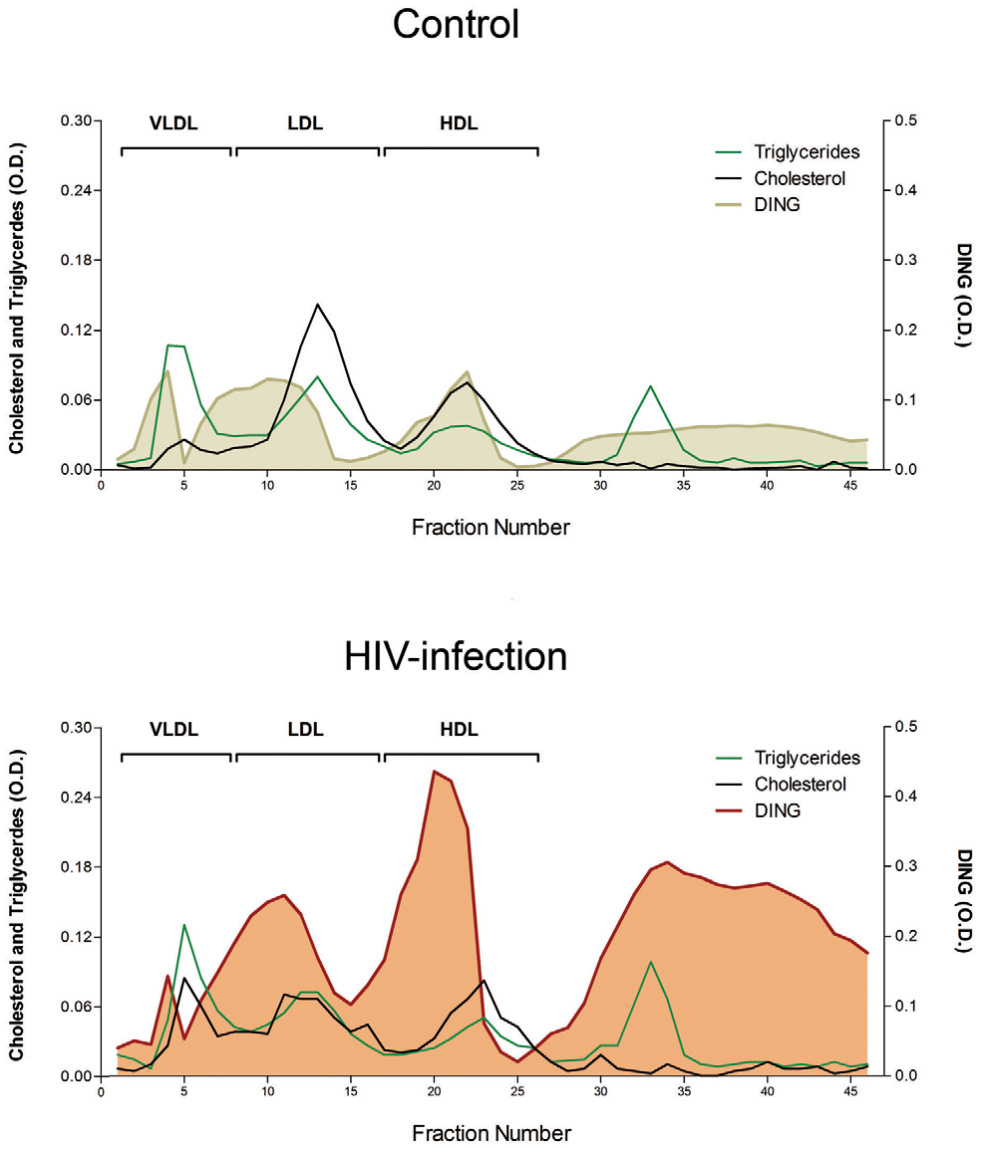
DING proteins distribution in lipoproteins fractions from HIV-infected and control pool. Pools of two HIV-infected patients and two controls were used in this experiment; results are represented as the optical densities at 450 nm.

In the control pool, DING proteins were eluted in HDL fractions, together with PON1, but also in IDL particles, an intermediate form between VLDL and LDL. A substantial amount of DING proteins was observed in the lipoprotein deficient serum (LPDS) from fractions 25 to 45, corresponding to soluble, free or DING proteins bound to albumin or other serum proteins. The HIV-infected pool showed a similar pattern of distribution, but with a global increase that was especially marked in the HDL and free DING proteins fractions (Fig. 3).

### Relationships between serum DING proteins concentration and the immunological and virological outcomes

**Figure 4:**
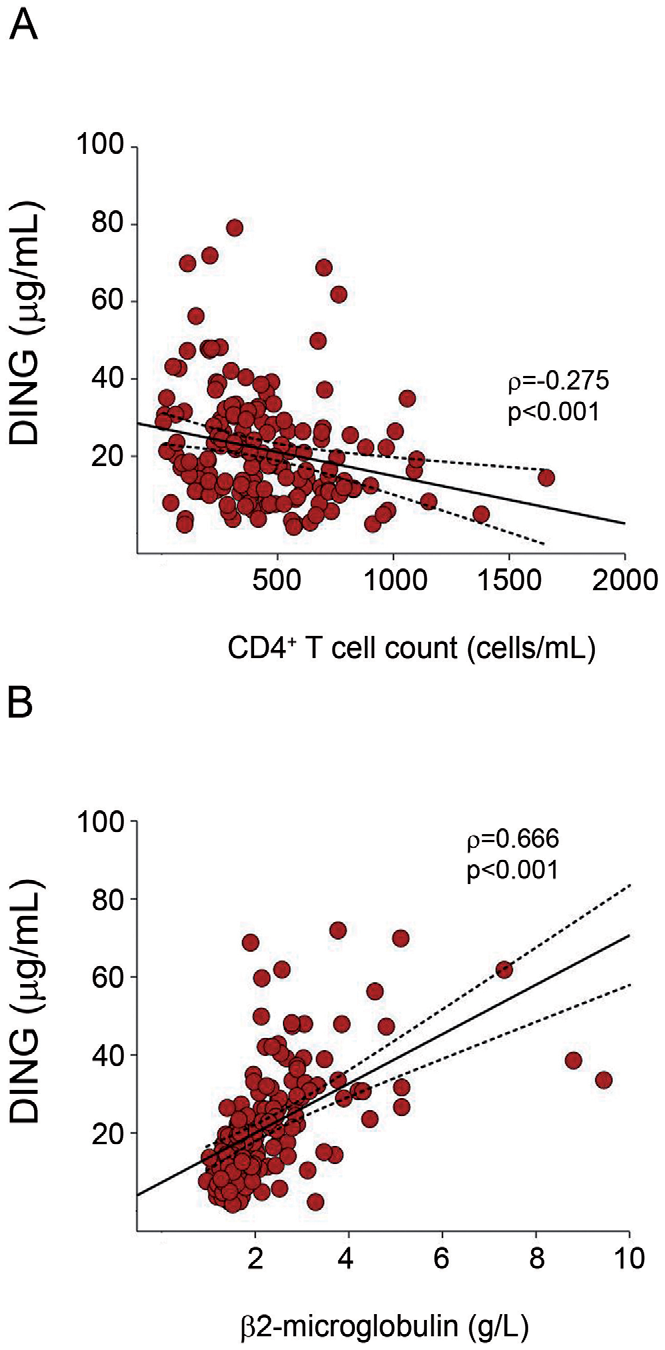
Relationship between DING proteins concentrations and some HIV markers. Correlation of DING proteins concentration with (**A**) CD4+ T cell counts and (**B**) β-2-microglobulin in HIV-infected patients.

DING proteins concentration was directly associated with viral load (*r* = 0.332; p<0.001). HIV-infected patients with a negative HIV-1 plasma viral load had significantly lower serum DING proteins concentration than those with a positive viral load [14.6 (2.7–48.0) vs. 24.1 (7.5–50.5) mg/l, respectively; p<0.001]. A highly significant inverse association was observed between serum DING proteins concentration and CD4+ T cell counts (*r* = −0.275; p<0.001) (Fig. 4A), and with the CD4+/CD8+ ratio (*r* = −0.270; p = 0.001). There was a highly significant linear direct relationship between serum DING proteins and β-2-microglobulin concentrations (*r* = 0.666; p<0.001; Fig. 4B). Co-infection with HCV was also associated with a significantly higher serum DING proteins concentration [21.0 (4.8–56.8) vs. 14.6 (3.5–44.5) mg/l, respectively; p = 0.016].

### Influence of treatments on serum DING proteins concentrations in HIV-infected patients

**Figure 5:**
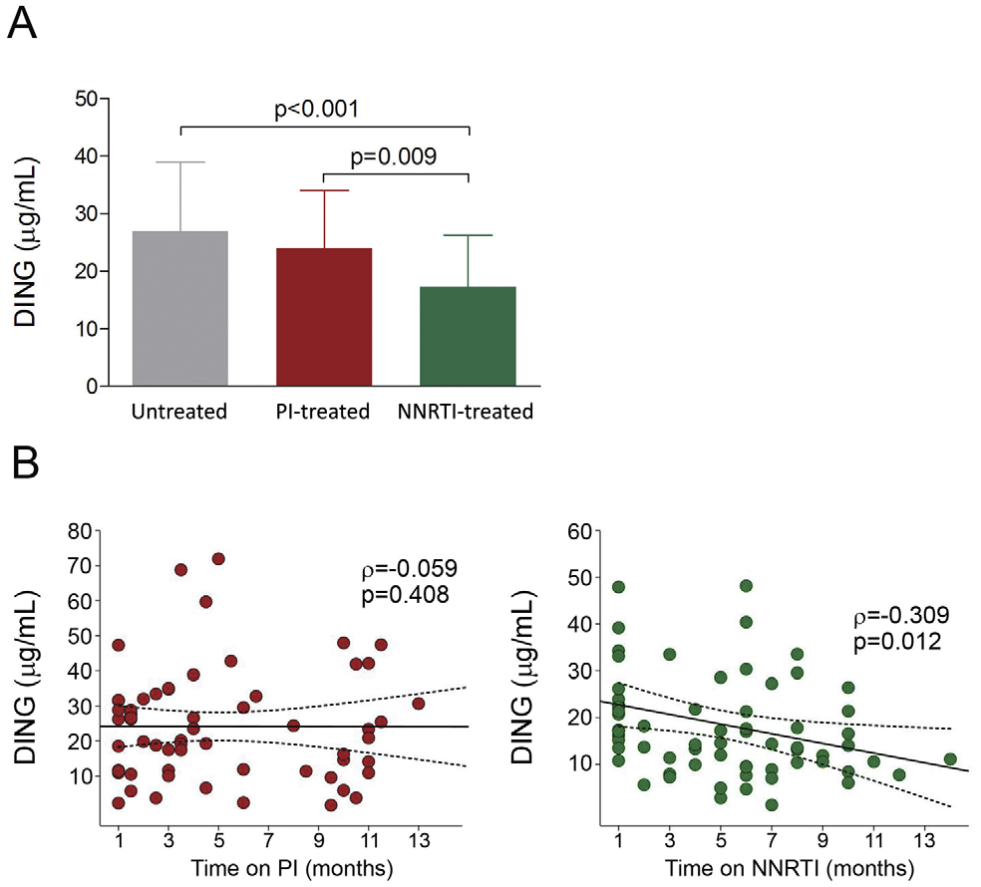
Influence of treatment on DING proteins concentration. (**A**) Effect of PIs and NNRTIs based treatment on DING proteins concentration compared to naives HIV-infected patients; results are represented as medians and IQRs. (**B**) Relationship between DING proteins concentration and type/duration of NNRTIs or PIs treatment.

Serum DING proteins concentration was significantly affected by the type of antiretroviral treatment followed. As shown in Fig. 5A, both untreated HIV-infected patients and patients treated with PIs had higher levels of DING proteins than those undergoing a NNRTIs-based antiretroviral treatment regimen [26.9 (17.4–38.9) and 24.0 (15.4–34.5) vs 17.2 (8.4–26.3) mg/l; respectively]. Moreover, we observed a significant inverse relationship between serum DING proteins concentration and the duration of the antiretroviral treatment with NNRTI (*r* = −0.309; p = 0.012), but not in HIV-infected patients receiving PIs (*r* = −0.059; p = 0.408) (Fig. 5B).

### *In vitro* HIV-infection of cell culture

**Figure 6:**
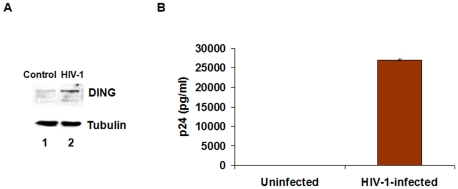
*In vitro* HIV-infection of cell culture and its impact on the expression of DING proteins. (**A**) Expression of DING proteins detected by western blot using anti-HPBP antibody in HIV-infected cells (right panel) or non infected control cells (left panel). (**B**) p24 antigen quantified 6 days post-infection using a p24-ELISA.

Infection of human PBMC with HIV-1 caused overproduction of DING proteins detected by monoclonal anti-HPBP antibody (Figure 6 A, top panel, lane 2) compared to uninfected cells (Lane 1). HIV-1 replication on the sixth day post-infection was also confirmed by p24 ELISA for the presence of HIV-1 p24 protein (Figure 6 B). Data from this experiment suggest that DING proteins seem to be expressed in response to HIV infection.

## Discussion

Recent studies on DING proteins have highlighted the anti-HIV potential of some members of this family [8], [17], [18], [20]. During this study, we have developed a homemade ELISA for the quantification of serum DING proteins. Using this method, we were able to measure for the first time the concentration of serum DING proteins in healthy people (7.9 (4.27–11.24) mg/l). In contrast, our results show that DING proteins concentration was increased in both serum and lipoproteins fractions of HIV-infected patients with respect to controls. This increase is likely to be a response of the organism to HIV infection. However, the pathophysiological significance of this increase remains unclear. In addition, cell cultures infected with HIV show also an overexpression of DING proteins. This overexpression was not related to treatment side effects since these cells were not treated with any antiretroviral medication.

Expression of DING proteins in response to HIV-infection has been previously reported by Simm et al. [32], [33]. Indeed, it has been shown that primary CD4+ T cells exposed to an attenuated form of HIV-1 (*Δvif* HIV-1) acquire resistance to the wild-type virus [32], [33]. Interestingly, these resistant cells were permissive to virus entry, reverse transcription of RNA, integration of the viral genome in the DNA of the cell, but were able to block transcription of HIV-1 genes [33]. Moreover, it has been shown that this phenotype was mediated via the production of a DING protein that they have named X-DING CD4 (formerly HIV resistance factor or HRF) [8]. Recently, it has been shown that HIV-exposure of these resistant cells causes a rapid up-regulation of X-DING CD4 at the mRNA level [34]. Given this potent anti-HIV activity, the author proposed that this factor may be considered as a part of an ancient defense mechanism of the organism against HIV [34], [35]. In fact, recent discoveries have shown that human cells are able to produce a so called “restriction factors” that interfere with HIV and other retroviruses replication. Particularly, APOBEC3, Tethrin, TRIM5, defensins and other antiretroviral factors has been shown to block HIV at different stages of its replication cycle [35], [36], [37], [38]. It has been proposed that these factors could be part of an ancient defense mechanism against HIV, which has otherwise evolved a “toolkit” to overcome their effects. From the known anti-HIV potential of some human DING representatives, it is not to be excluded that DING proteins may make part of this unusual defense mechanism against HIV or other viruses.

We have also observed a significant direct relationship between DING proteins and PON1 concentration in HIV-infected patients. This increase may be explained by the recently described association between PON1 and the DING protein HPBP. Indeed, in human plasma HPBP and PON1 were found to be tightly associated, and it has been shown that this association is required for the stability of each other [13], [14]. However, an inverse relationship was observed between the concentration of DING proteins and both PON1 lactonase and paraoxonase activities. In fact, it is known that PON1 activities are greatly modulated by its molecular environment on HDL particles. In addition, PON1 need to be bound to HDL for its optimum activity [39]. Since PON1 can be tightly associated with HPBP on HDL surface, a modulation of its activity by HPBP seems to be possible.

Our experiments on the fractionation of lipoproteins give new information about DING proteins localization. Our results show that DING proteins are localized mainly on HDL and IDL particles, an intermediate form between VLDL and LDL. Given the association between HPBP and the HDL-associated PON1, it seems that DING proteins found on IDL are directly linked or in association with other proteins. We found also a substantial amount of DING proteins in a free form in the LPDS fractions. This finding indicates that DING proteins may also be present in free form as circulating proteins, maybe in association with other proteins, but not linked to any lipoprotein particle. Identification of these putative partners will be helpful to better understand their physiological functions.

We investigated the relationship between DING proteins concentration and several HIV markers like CD4 counts, β-2-microglobulin and viral load. Our results show an inverse relationship between DING proteins concentration and CD4 counts. Interestingly, a very strong direct association with β-2-microglobulin has been observed. It is worth to be noted that many studies consider β-2-microglobulin as an effective marker of HIV disease progression [29], [40]. Combined with our results from ROC curve analysis, we suggest that serum DING proteins dosage may be considered as a potential predictor factor of HIV. In addition, DING proteins concentration was directly associated with the viral load. Interestingly, HIV-infected patients with a negative viral load kept a relatively high concentration of DING proteins compared to controls. It seems that once infected, the organism stay producing relatively high amounts of DING proteins. The molecular mechanisms responsible of this response are still unknown and need to be further studied.

Another intriguing finding was the effect of treatment on the DING proteins concentration. In fact, in contrary to antiproteases, treatment with non-nucleoside reverse transcriptase inhibitors was correlated with a decrease of DING proteins concentration. It seems possible that overexpression of DING proteins occurs between these two steps targeted by the therapy. May viral genome integration induce DING proteins overexpression? Indeed, many studies have shown that HIV infection can modulate the expression of several genes [41], [42]. Hence, a direct implication of HIV on the expression of DING proteins is not to be excluded.

Our results, together with the presence in humans of DING proteins with anti-HIV activity (HPBP and X-DING CD4), raise a major question of why this increase is not correlated with a resistance to HIV infection. One possibility is that DING proteins are not “bioavailable” and cannot inhibit HIV when associated with other proteins. For instance, HPBP is not active against HIV when associated with PON1 [17]. So we can think about a mechanism in which DING proteins need to be released from their partners to be able to exercise their functions.

In summary, results from this study show that human organism reacts to HIV infection by an overexpression of DING proteins. This overexpression was also observed in the in vitro infection of cell cultures, ruling out any influence of treatment on this increase. This may have a prognostic value to assess HIV infection in newly or already treated HIV-infected patients. Anyway, DING proteins stay enigmatic, and further studies are needed to deepen our knowledge on this family of proteins.

## Supporting Information

Table S1 **General characteristics of the HIV-infected patients (n = 207).**
(DOC)
