# Supplementary material for: The Level of DING Proteins Is Increased in HIV-Infected Patients: In Vitro and In Vivo Studies
Source: PLoS One. 2012 Mar 9;7(3):e33062. doi: 10.1371/journal.pone.0033062 (PMC3302901; doi:10.1371/journal.pone.0033062)
Supplement: Table S1 — General characteristics of the HIV-infected patients (n = 207). (DOC) [file pone.0033062.s001.doc]

| Table S1General characteristics of the HIV-infected patients (n = 207) | |
| --- | --- |
| Characteristics | Value |
| Age, years | 38 (7) |
| Gender, male (%) | 139 (67.1) |
| BMI, kg/m^2^ | 23.1 (3.2) |
| Conventional cardiovascular disease risk factors, n (%) |  |
| Current smoker | 163 (78.7) |
| Hypertension | 18 (8.7) |
| Abnormal fasting glucose | 17 (8.2) |
| Dyslipidemia | 75 (36.2) |
| Risk factors for HIV infection, n (%) |  |
| Intravenous drug use | 117 (56.5) |
| Male homosexual contact | 25 (12.1) |
| Heterosexual contact | 60 (29.0) |
| Years since HIV diagnosis | 5.4 (3.3) |
| Baseline CD4+ T count, cells/mm^3^ * | 444.4 (286.6) |
| Baseline CD8+ T count, cells/mm^3^ * | 1084.3 (580.7) |
| Ratio CD4+ / CD8+ * | 0.41 (0.33) |
| Viral load <200 copies/mL, n (%) | 81 (39.1) |
| AIDS-related disease, n (%) | 68 (32.8) |
| Hepatitis C virus co-infection, n (%) | 122 (58.9) |
| Presence of lipodystrophy, n (%) | 45 (21.7) |
| Antiretroviral therapy, n (%) |  |
| Untreated | 52 (25.1) |
| NNRTI | 84 (40.6) |
| PI | 71 (34.3) |
| NRTI | 129 (62.3) |
| Treated with statins, n (%) | 4 (1.9) |
| Treated with fibrates, n (%) | 17 (8.2) |
| Quantitative variables are reported as means and SD (in parentheses). Qualitative variables are reported as n and % (in parentheses).  *These parameters were analyzed in 177 patients.  NRTI: nucleoside reverse transcriptase inhibitor; NNRTI: non-nucleoside reverse transcriptase inhibitor; PI: protease inhibitor. | |
